# Clinical Characteristics of *Corynebacterium ulcerans* Infection, Japan

**DOI:** 10.3201/eid2908.220058

**Published:** 2023-08

**Authors:** Akihiko Yamamoto, Toru Hifumi, Manabu Ato, Masaaki Iwaki, Mitsutoshi Senoh, Akio Hatanaka, Shinichi Nureki, Yoshihiro Noguchi, Tomoko Hirose, Yukihiro Yoshimura, Takaaki Urakawa, Shiro Hori, Hiroto Nakada, Tomomasa Terada, Tomoko Ishifuji, Hisayo Matsuyama, Takahiro Kinebuchi, Atsuhito Fukushima, Koji Wake, Ken Otsuji, Takeru Endo, Hirokazu Toyoshima, Ikkoh Yasuda, Takeshi Tanaka, Naoki Takahashi, Kensaku Okada, Toshimasa Hayashi, Taizo Kusano, Minami Koriyama, Norio Otani, Motohide Takahashi

**Affiliations:** National Institute of Infectious Diseases, Tokyo, Japan (A. Yamamoto, M. Ato, M. Iwaki, M. Senoh);; St. Luke’s International Hospital, Tokyo (T. Hifumi, N. Otani);; Ageo Central Genral Hospital, Saitama, Japan (A. Hatanaka);; Oita University Faculty of Medicine, Oita, Japan (S. Nureki);; International University of Health and Welfare School of Medicine, Chiba, Japan (Y. Noguchi);; Japanese Red Cross Otsu Hospital, Shiga, Japan (T. Hirose);; Yokohama Municipal Citizen’s Hospital, Kanagawa, Japan (Y. Yoshimura);; Tsuruoka Municipal Shonai Hospital, Yamagata, Japan (T. Urakawa);; Japan Community Healthcare Organization Ritsurin Hospital, Kagawa, Japan (S. Hori);; Holon Toriizaka Clinic, Tokyo (H. Nakada);; Tokushima Prefectural Central Hospital, Tokushima, Japan (T. Terada);; Itabashi Medical System Tokyo–Katsushika General Hospital, Katsushika, Tokyo (T. Ishifuji);; Kawakita General Hospital, Suginami, Tokyo (H. Matsuyama);; Furano Hospital, Hokkaido, Japan (T. Kinebuchi);; Dokkyo Medical University, Tochigi, Japan (A. Fukushima, K. Wake);; Hospital of University of Occupational and Environmental Health, Fukuoka, Japan (K. Otsuji, T. Endo);; Japanese Red Cross Ise Hospital, Mie, Japan (H. Toyoshima);; Fukushima Medical University, Fukushima, Japan (I. Yasuda);; Nagasaki University Hospital, Nagasaki, Japan (T. Tanaka);; Kimitsu Chuo Hospital, Chiba (N. Tanaka);; Tottori University Hospital, Tottori, Japan (K. Okada);; Maebashi Red Cross Hospital, Gunma, Japan (T. Hayashi);; Chiba Children’s Hospital, Chiba (T. Kusano);; Chiba Rousai Hospital, Chiba (M. Koriyama);; Kumamoto Health Science University, Kumamoto, Japan (M. Takahashi)

**Keywords:** *Corynebacterium ulcerans*, bacteria, bacterial infections, companion animals, diphtheria, Japan, respiratory infections

## Abstract

*Corynebacterium ulcerans* is a closely related bacterium to the diphtheria bacterium *C. diphtheriae*, and some *C. ulcerans* strains produce toxins that are similar to diphtheria toxin. *C. ulcerans* is widely distributed in the environment and is considered one of the most harmful pathogens to livestock and wildlife. Infection with *C. ulcerans* can cause respiratory or nonrespiratory symptoms in patients. Recently, the microorganism has been increasingly recognized as an emerging zoonotic agent of diphtheria-like illness in Japan. To clarify the overall clinical characteristics, treatment-related factors, and outcomes of *C. ulcerans* infection, we analyzed 34 cases of *C. ulcerans* that occurred in Japan during 2001–2020. During 2010–2020, the incidence rate of *C. ulcerans* infection increased markedly, and the overall mortality rate was 5.9%. It is recommended that adults be vaccinated with diphtheria toxoid vaccine to prevent the spread of this infection.

## Abstract 2

Incidence has been increasing markedly, and the case-fatality rate is 5.9%.

Diphtheria is an upper respiratory tract illness caused by toxin-producing *Corynebacterium diphtheriae* bacteria, and it is characterized by sore throat, fever, and formation of a pseudomembrane on the tonsils, pharynx, or both, along with nasal discharge. *C. diphtheriae* can also infect the skin, causing open sores or ulcers. However, diphtheria skin infections rarely result in any other severe disease (*1*). *C. ulcerans* is a closely related bacterium to *C. diphtheriae*, and some strains produce toxins that are very similar to diphtheria toxin (*2*,*3*). *C. ulcerans* is widely distributed in the environment and is considered one of the most harmful pathogens to livestock and wildlife. This bacterium can cause cutaneous inflammation, including mastitis, in dairy cows (*4*–*6*). *C. ulcerans* has been increasingly recognized as an emerging zoonotic agent of diphtheria-like illness in the world (*7*–*18*).

Infections caused by these 2 bacteria are difficult to distinguish clinically, and the World Health Organization (WHO) treats infections caused by toxin-producing *C. ulcerans* as part of the diphtheria case definition (*19*). *C. diphtheriae* is thought to be transmitted only among humans, but *C. ulcerans* can be transmitted to humans by nonhuman mammals and thus should be treated as a zoonosis (*7*–*18*). Dogs and cats as companion animals are considered the major causes of transmission to humans. Although there have been several reports of individual cases of *C. ulcerans* infection (*20**–**24*), information on clinical features, treatment-related factors, and outcomes is limited. In this study we elucidate the clinical features, treatment-related factors, and outcomes of *C. ulcerans* infection cases in Japan during 2001–2020.

## Methods

This study was a retrospective, observational, national survey of *C. ulcerans* infections in Japan since the first reported case of this infection (*25*). The data acquisition period was 20 years, from February 2001 through December 2020. The institutional review board of St. Luke’s International Hospital (Tokyo, Japan) approved this cross-sectional, survey-based study (approval no. 19-R055).

### Patients and Setting

The National Institute of Infectious Disease (NIID) has comprehensively organized research and controlled clinical practice in *C. ulcerans* infectious diseases in Japan. However, in Japan, *C. ulcerans* infection is not included in the diphtheria case definition, nor is it required to be reported in all cases, so there is no obligation to report. However, because *C. ulcerans* produces diphtheria toxin, it has clinical manifestations similar to those caused by *C. diphtheriae*, for which all cases must be reported in Japan (*19*). Therefore, Japan’s Ministry of Health, Labour and Welfare (MHLW) urged health management departments and hospitals throughout the country to call attention to the need to identify the causative bacterium in patients showing clinical symptoms similar to diphtheria. MHLW has also published diagnostic criteria for *C. ulcerans* to assist clinicians in classifying *C. diphtheriae* and *C. ulcerans* (*26*). Under those circumstances, information from doctors who treated patients with suspected diphtheria symptoms and requests for pathogen diagnosis were sent to NIID. Therefore, the data included in this analysis came from attending physicians who, at the time of care, chose to investigate and report cases as *C. ulcerans* infections.

### Data Collection

The following parameters were recorded: age; sex; date of infection; location of patient’s origin; whether there was a companion animal; whether there was any interaction with animals, such as breeding livestock animals, or whether the patient had lived in an environment involving contact with animals; presence or absence of bacterial isolation from patients and related animals; and clinical symptoms (throat pain, nasal discharge, pseudomembrane, fever, headache, dyspnea, hoarseness, and abscess). In addition, we collected data on vital signs (heart rate, systolic blood pressure, temperature, and respiratory rate), laboratory data (leukocyte counts, platelet counts, creatinine kinase levels, C-reactive protein [CRP] levels), types of antibiotics administered, presence or absence of administration of diphtheria antitoxin, and outcomes (days of hospitalization, days of mechanical ventilation, and survival or death).

### Diagnosis of *C. ulcerans* Infection

The diagnostic criteria for the cases collected in this study used the *C. ulcerans* diagnostic criteria of MHLW (*26*). Accordingly, several conditions must be met: the infection manifests the same clinical symptoms as respiratory diphtheria with intractable pharyngeal pseudomembrane formation, and gram-positive rods are isolated from local areas, such as the pharynx and nasal cavity, and identified as *C. ulcerans*; the isolated bacterium is *C. ulcerans* alone, or *C. ulcerans* is the main component; detection of the diphtheria toxin gene and its toxin activity have been confirmed from this isolated strain by a functional test (i.e., an Elek test or equivalent test) (*1*,*26*); and cutaneous signs and symptoms are present and *C. ulcerans* is identified as the causative agent of local lymphadenopathy and abscesses (*27*,*28*).

### Definitions of Symptoms

*C. ulcerans* infections are classified into respiratory and nonrespiratory manifestations. We defined respiratory symptoms as dyspnea, hoarseness, sore throat, cough, fever, and (occasionally) white pseudomembrane of the nasopharynx and laryngeal vestibule. Nonrespiratory symptoms were defined as skin infections and abscesses, or symptoms in patients who did not show respiratory symptoms. We further classified both types of symptoms as mild (resolving on outpatient visits), moderate (requiring hospitalization), or severe (requiring hospitalization and further ventilator support). The definitions of all cases included in this study were those we described previously as consistent with diagnosis of *C. ulcerans* infection.

### Treatment of *C. ulcerans* Infection

For treatment of *C. ulcerans* infection, administration of antibiotics to which *C. ulcerans* is susceptible, such as macrolides and penicillins, is effective. In severe cases, symptomatic treatment for diphtheria pneumonia and administration of diphtheria antitoxin are effective for ventilated patients. The antitoxin used for *C. ulcerans* infection and for diseases caused by *C. diphtheriae* is delivered from the nearest national stockpile. However, depending on the distance, sometimes immediate delivery cannot be achieved.

### Primary Data Analysis

We compared patients’ characteristics, treatment-related factors, and outcomes between the respiratory symptoms group and nonrespiratory symptoms group by using the Mann–Whitney U test or Fisher exact test, as appropriate. We used quantitative properties in the calculation basically as they are and quantified qualitative properties by scoring and then analyzed them. In the respiratory symptoms group, we compared mild, moderate, and severe cases. Regarding the collection of clinical data, we did not impute missing data. We performed statistical analysis by using JMP Pro statistical software version 14 (SAS Institute, https://www.sas.com). We considered 2-sided p values <0.05 to be statistically significant.

## Results

### Demographic and Clinical Characteristics of Patients

**Figure 1:**
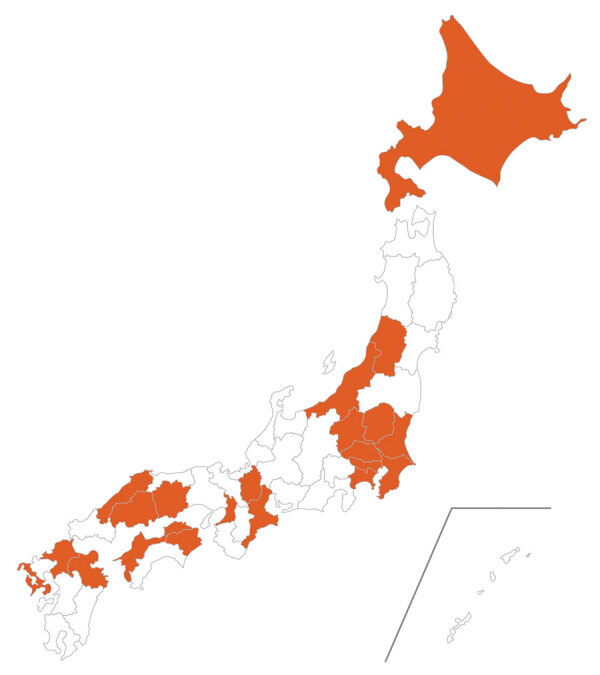
Prefectures containing outbreak areas of *Corynebacterium ulcerans* infection (orange), Japan, 2001–2020. Inset map shows the Nansei Islands, an archipelago in the southwestern part of Japan.

**Figure 2:**
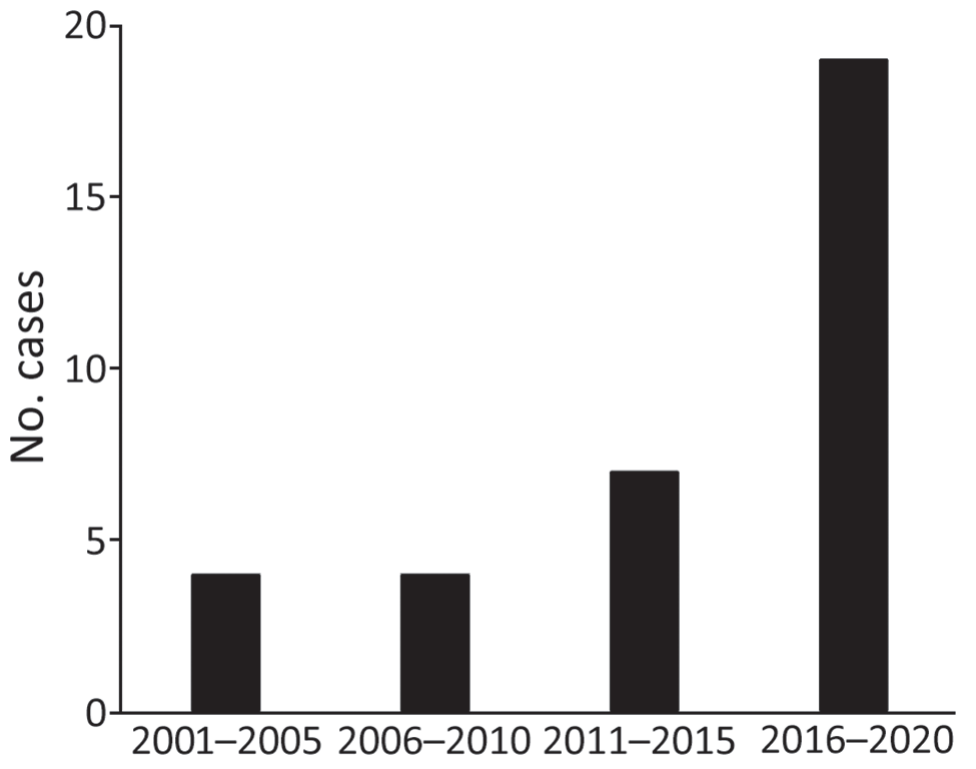
Increase in the number of *Corynebacterium ulcerans* infection cases, by 5-year period, Japan, 2001–2020.

A total of 34 patients from 34 hospitals were identified during the 20-year study period (Appendix Table). The reports of *C. ulcerans* infections came from a wide range of areas, and there was no regional bias (Figure 1). Furthermore, when we compared the number of *C. ulcerans* cases every 5 years, we found the number of cases during 2001–2010 was stable (4 total cases), but the number of cases during 2011–2015 was 7 and during 2016–2020 was 19. Therefore, compared with the number of cases during 2001–2010, the number during 2011–2015 was 1.75 higher and during 2016–2020 was 4.75 times higher (Figure 2).

The symptoms of *C. ulcerans* infection were respiratory in 23 (67.7%) patients and nonrespiratory in 11 (32.3%) (Table 1). The median age of patients was 58 years, and 61.3% of patients were women. Almost all patients (97.1%) had contact with animals. The mortality rate was 5.9%. 

**Table 1:** Characteristics of patients with *Corynebacterium ulcerans* infection, Japan, 2001–2020*

Characteristic	All cases, N = 34†	Respiratory cases, n = 23‡	Nonrespiratory cases, n = 11§	p value
Median age, y	58 (50–71)	64 (54–72)	38 (21–61)	0.03
Sex				
M	12 (38.7)	7 (31.8)	5 (55.6)	0.25
F	19 (61.3)	15 (68.2)	4 (44.4)	0.19
Relationship with animals¶	33 (97.1)	22 (95.7)	11 (100)	1.00
Vital signs on admission				
Heart rate, beats/min	100 (88–112)	101 (97–114)	85 (85–85)	0.13
Systolic blood pressure, mm Hg	125 (107–146)	130 (108–147)	107 (107–107)	0.32
Body temperature, °C	38 (37.4–38.5)	38 (37.4–38.4)	38.4 (35.5–38.5)	0.94
Respiratory rate, breaths/min	20 (16–26)	18 (16–28)	21 (21–21)	0.61
Laboratory data				
Leukocytes, cells/mm^3^	13,800 (9,325–18,900)	14,800 (10,850–21,700)	10,500 (7,775–12,700)	0.07
Platelets, × 10^4^/mm^3^	26.3 (22.1–27.2)	25.1 (19.9–34.1)	26.6 (26.3–26.8)	0.51
Creatine, mg/dL	0.75 (0.66–1.16)	0.75 (0.67–1.24)	0.59 (0.38–0.80)	0.34
C-reactive protein, mg/dL	6.1 (3.7–16.8)	10.8 (4.7–21)	3.9 (2.3–5.7)	0.07
Treatment antibiotic (no. cases)				
Penicillins	Penicillin G (2), sulbactam/ampicillin (9), piperacillin (5)	Penicillin G (1), sulbactam/ampicillin (8), piperacillin (4)	Penicillin G (1), sulbactam/ampicillin (1), piperacillin (1)	
Macrolides	Erythromycin (9), clarithromycin (6), azithromycin (6), clindamycin (1)	Erythromycin (6), clarithromycin (5), azithromycin (5), clindamycin (1)	Erythromycin (3), azithromycin (1), clarithromycin (1)	
Cephalosporins	Cephepime (1), cefazolin (1), ceftriaxone (1)	Cephepime (1), ceftriaxone (1)	Cefazolin (1)	
Quinolones	Levofloxacin (3)	Levofloxacin (2)	Levofloxacin (1)	
Other	Meropenem (3), faropenem (1), minocycline (1)	Meropenem (3)	Faropenem (1), minocycline (1)	
Diphtheria antitoxin	4 (11.8)	4 (17.4)	0	0.28
Classification of respiratory symptoms				
Mild	8	6	2	
Moderate	16	7	9	
Severe	10	10	0	
Outcome				
Hospital days	10 (3–30)	13 (4–31)	9 (0–26)	0.41
Ventilator days	0 (0–6)	2 (0–12)	0	0.04
Deaths#	2 (5.9)	2 (8.7)	0	1.00

*Data are medians (interquartile range) for continuous variables and no. (%) for categorical variables. †Missing data for all cases: age (n = 3), sex (n = 3), heart rate (n = 27), systolic blood pressure (n = 27), body temperature (n = 19), respiratory rate (n = 27), leukocytes (n = 17), platelets (n = 26), creatine (n = 23), C-reactive protein (n = 17), treatment (n = 10), hospital days (n = 8), ventilator days (n = 9). ‡Missing data for respiratory cases: age (n = 1), sex (n = 1), heart rate (n = 17), systolic blood pressure (n = 17), body temperature (n = 11), respiratory rate (n = 17), pseudomembrane (n = 1), leukocyte (n = 10), platelets (n = 17), T-bilirubin (n = 17), creatine (n = 14), C-reactive protein (n = 10), treatment (n = 5), hospital days (n = 3), ventilator days (n = 4). §Missing data for nonrespiratory cases: age (n = 2), sex (n = 2), heart rate (n = 10), systolic blood pressure (n = 10), body temperature (n = 8), respiratory rate (n = 10), leukocytes (n = 7), platelets (n = 10), T-bilirubin (n = 11), creatine (n = 9), C-reactive protein (n = 7), treatment (n = 5), hospital days (n = 5), ventilator days (n = 5). ¶Indicates the presence of animals in the patient’s living environment. #All deaths were in cases for which respiratory symptoms were classified as severe.

### Details of *C. ulcerans* Infection Cases

**Figure 3:**
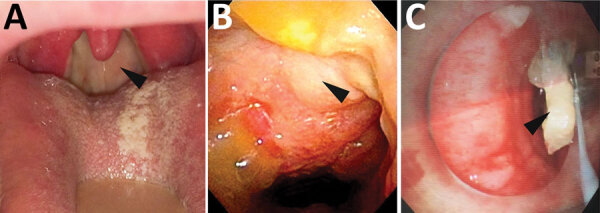
Endoscopic images of the pharynx and bronchi of patients with *Corynebacterium ulcerans* infection, Japan, 2001–2020. A, B) Posterior wall of the pharynx has a yellowish white pseudomembrane. Arrows indicate the white pseudomembrane attached to the pharynx (case no. 21, from Dr. Toyoshima, Japanese Red Cross Ise Hospital, Mie, Japan). C) Pseudomembrane on the bronchi. Arrows indicate the pseudomembrane attached to the bronchi (case no. 29, from Dr. Hayashi, Maebashi Red Cross Hospital, Gunma, Japan).

**Figure 4:**
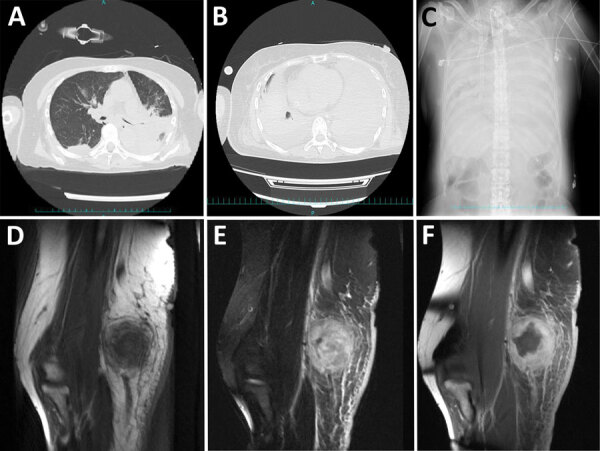
Computed tomography, radiograph, and magnetic resonance imaging results for patients with *Corynebacterium ulcerans* infection, Japan, 2001–2020. A–C) Chest computed tomography images and radiograph of patients with severe respiratory symptoms. Atelectasis noted at admission (A, top) and after exacerbation of symptoms (B, bottom) (case no. 29, from Dr. Hayashi, Maebashi Red Cross Hospital, Gunma, Japan). Spread of atelectasis noted on chest radiograph taken at the time of exacerbation of symptoms (C) (case no. 29, also from Dr. Hayashi, Maebashi Red Cross Hospital). D–F) Magnetic resonance imaging of an elbow abscess. Magnetic resonance imaging T1-weighted images show equal brightness to the muscles (D), fat-suppressed T2-weighted image by short-tau inversion recovery method show unevenly high brightness (E), and contrast-enhanced T1-weighted images show a mass whose margins are contrast-enhanced (F) (case no. 10, from Dr. Urakawa, Tsuruoka Municipal Shonai Hospital, Yamagata, Japan).

We divided the clinical characteristics of *C. ulcerans* patients into respiratory and nonrespiratory groups. The characteristics of respiratory symptoms include formation of pseudomembrane in addition to dyspnea, hoarseness, sore throat, and fever. In general, among the patients evaluated in our study, the pseudomembrane was often attached to the nasopharynx (Figure 3, panels A, B; Figure 4). Moreover, in severe cases, bronchoscopy showed a pseudomembranous material obstructing the bronchi (Figure 3, panel C). 

Patients with pseudomembranes with bronchial obstruction were characterized by atelectasis (i.e., when part or all of the lung was devoid of air and collapses) on radiographs (Figure 4, panel A). A common cause of atelectasis was bronchial obstruction. Atelectasis spreads throughout the lungs as symptoms worsen (Figure 4, panels B, C). As lung function declines, ventilators and extracorporeal membrane oxygen therapy are required to save the patient’s life. Complications after *C. ulcerans* treatment have been reported (*29*); in that particular case, the patient reported dyspnea, and a thick pseudomembrane was found in the larynx. *C. ulcerans* producing diphtheria toxin was detected in the pseudomembranes. Antibiotic treatment improved airway symptoms, but sudden cardiac arrest occurred, followed by dyspnea and seizures. Afterward, the patient’s general condition stabilized, but she remained unconscious.

In contrast, nonrespiratory *C. ulcerans* patients evaluated in our study had local lymph node abscesses near the trauma, parotid abscesses, axillary abscesses, cervical lymph node abscesses, plantar skin ulcers, subcutaneous abscesses, and mandibular abscesses. Symptoms such as abscess, thigh abscess, and purulent lymphadenitis in the right neck were observed (Appendix). MRI images of a patient’s elbow showed abscesses in the axillary and parotid lymph nodes (Figures 4, panels E–F).

### Comparing Clinical Characteristics between Respiratory and Nonrespiratory Symptoms Groups

Patients in the respiratory symptoms group were significantly older than patients in the nonrespiratory group (64 [interquartile range (IQR) 54–72] years vs. 38 [IQR 21–61] years; p = 0.03). When we compared the 3 severity classifications for respiratory and nonrespiratory symptoms, we found the group with respiratory symptoms had 6 patients with mild, 7 patients with moderate, and 10 patients with severe symptoms. In contrast, in the nonrespiratory symptom group, there were 5 mild cases, 6 moderate cases, and 0 severe cases. The differences in the number of mild, moderate, and severe cases of symptom severity in the 2 groups were significant (p<0.01) (Table 1). Leukocyte counts and CRP levels were relatively higher in the respiratory group than in the nonrespiratory group (p = 0.07 for both) (Table 1).

### Comparing Clinical Characteristics among the 3 Severity Groups

Within the group showing respiratory symptoms, we compared clinical characteristics for the mild, moderate, and severe subgroups (Table 2). Among the respiratory group patients with pseudomembrane, 5 had mild cases, 6 had moderate cases, and 10 had severe cases. Among cases with respiratory symptoms, pseudomembrane-positive patients accounted for 83.3% of mild cases, 85.7% of moderate cases, and 100% of severe cases. Laboratory data showed a significant difference among the 3 subgroups in CRP levels (4.7 mg/dL [IQR 0.9–6.1 mg/dL] in mild, 7.7 mg/dL [IQR 1.8–12.6 mg/dL] in moderate, and 21 mg/dL [IQR 11.7–25.4 mg/dL] in severe cases; p = 0.02). Macrolide antibiotics, which are effective for *C. ulcerans*, were mainly used for mild cases. As the severity increased, many additional antibiotics, such as penicillin, cephalosporin antibiotics, and quinolone, were used. Regarding the length of hospital stay, we observed a significant difference between moderate cases (7 [IQR 7–10] days) and severe cases (29 [IQR 20–56] days; p<0.01). Diphtheria antitoxin was administered only to 4 severe case-patients (cases 5, 24, 29, and 33). 

**Table 2:** Comparison of the subgroups of patients with *Corynebacterium ulcerans* infection with mild, moderate, and severe respiratory symptoms, Japan, 2001–2020*

Characteristic	Mild symptoms, n = 6†	Moderate symptoms, n = 7‡	Severe symptoms, n = 10§	p value
Age, y	54 (28–61)	62 (51–76)	67 (62–72)	0.07
Sex				
M	3 (60.0)	3 (42.9)	1 (10.0)	0.11
F	2 (40.0)	4 (57.1)	9 (90.0)	0.29
Vital signs on admission				
Body temperature, °C	37 (36.6–37.4)	38 (37.6–38.8)	38 (37.5–38.7)	0.14
Pseudomembrane	5 (100)	6 (85.7)	10 (100)	0.33
Laboratory data				
Leukocytes, cells/mm^3^	9,500 (6,700–14,800)	14,350 (10,363–23,550)	18,900 (13,400–22,600)	0.26
C-reactive protein, mg/dL	4.7 (0.9–6.1)	7.7 (1.8–12.6)	21 (11.7–25.4)	0.02
Treatment antibiotic (no. cases)				
Penicillins	None	Penicillin G (1), sulbactam/ampicillin (2), piperacillin (2)	Sulbactam/ampicillin (6), piperacillin (2)	
Macrolides	Erythromycin (1), clarithromycin (2)	Erythromycin (3), clarithromycin (3), azithromycin (2)	Erythromycin (2), azithromycin (3), clindamycin (1)	
Cephalosporins		Ceftriaxone (1)		
Quinolones		Levofloxacin (2)		
Other			Meropenem (3)	
Diphtheria antitoxin	0	0	4 (40.0)	0.04
Outcome				
Hospital days	0	7 (7–10)	29 (20–56)	<0.01
Ventilator days	0	0	12 (5–42)	<0.01
Deaths	0	0	2 (20.0)	0.24

*Data are medians (interquartile range) for continuous variables and no. (%) for categorical variables. †Missing data for mild cases: age (n = 1), sex (n = 1), heart rate (n = 6), systolic blood pressure (n = 6), body temperature (n = 4), respiratory rate (n = 6), pseudomembrane (n = 1), leukocytes (n = 3), platelets (n = 6), C-reactive protein (n = 3), treatment (n = 3), hospital days (n = 3), ventilator days (n = 3). ‡Missing data for moderate: heart rate (n = 5), systolic blood pressure (n = 5), body temperature (n = 3), respiratory rate (n = 5), leukocytes (n = 3), platelets (n = 5), T-bilirubin (n = 5), creatine (n = 4), C-reactive protein (n = 3), treatment (n = 1). §Missing data for severe: heart rate (n = 6), systolic blood pressure (n = 6), body temperature (n = 4), respiratory rate (n = 6), leukocytes (n = 4), platelets (n = 6), T-bilirubin (n = 6), creatine (n = 5), C-reactive protein (n = 4), treatment (n = 1), ventilator days (n = 1).

Two deaths from *C. ulcerans* infection occurred among the severe case-patients (20%; cases 5 and 18). Case-patient 5 was administered 5,000 IU of diphtheria antitoxin on her second day of hospitalization. *C. ulcerans*, which had been detected in the pseudomembrane, became negative in culture 1 week later, but the patient died on the 21st day of hospitalization without improvement in her severe pneumonia. Case-patient 18 was not administered antitoxin. She was administered antibiotics but died on the third day of her hospitalization from severe dyspnea caused by a pseudomembrane obstructing her airway (*30*). 

## Discussion

To compare our findings to those from other countries, we reviewed reports on diphtheria from the United Kingdom (*31*,*32*) and Belgium (*33*). Because of the history of the diphtheria pandemic in Eastern Europe in the late 1980s, surveillance of reports of *C. diphtheriae* infection are still underway in Europe, and cases continue to be identified. Therefore, the literature cases from this region during that period also contain reports of disease caused by the diphtheria toxin–producing *C. diphtheriae* and *C. ulcerans* (*31*). In Japan, the most recent case report of *C. diphtheriae* infection was in 2000 (*34*), and since then, the number of *C. ulcerans* infections have been increasing, as shown in our study (Figure 2). The annual trend in the number of cases of *C. ulcerans* infection in the United Kingdom has also increased over the past few decades (*31*,*32*). In contrast, in Belgium, the number of cases reported during 2010–2017 hardly increased (*33*) and has remained fairly constant.

When we compared the age of patients with *C. ulcerans* infection, we found that in the United Kingdom, 60% of those affected are <15 years of age, whereas in Belgium, 90% of those affected are >45 years of age. In Japan, as in Belgium, 80% of those infected are >45 years of age. The sex ratio of patients showed similar trends in all countries; women accounted for 75% of infections in the United Kingdom, 77% in Belgium, and 67% in Japan. Martini et al. (*33*) argued that women are more likely to be patients because they tend to have more contact with companion animals than men.

When we compared transmission routes of *C. ulcerans*, we found that in the past in the United Kingdom, infections were mainly caused by cattle and poorly sterilized dairy products, but in recent years, infections have been mainly caused by companion animals such as cats and dogs. The same trends occurred in Belgium and Japan. The change over time in the source of *C. ulcerans* infection in the United Kingdom indicates that this infection is not limited to persons involved in livestock farming and that the general public can become infected (*32*). We speculate that this change contributed to the recent increase in *C. ulcerans* infections in the United Kingdom. Because *C. ulcerans* infections in countries such as the United Kingdom, Belgium, and Japan are suspected to be transmitted from companion animals, not only physicians but also veterinarians who examine companion animals should be informed about *C. ulcerans* infection (*8*,*11*,*16*).

Regarding the prognosis of *C. ulcerans* infection, mortality rates were 6% in the United Kingdom during 1986–2017 (*31*,*32*) and 5.9% in Japan during 2001–2020. No deaths from *C. ulcerans* infections were reported in Belgium during 2010–2017 (*33*). Further details of the course of fatal cases of *C. ulcerans* infection in the United Kingdom show that all of the fatal cases were in women >70 years of age who had respiratory symptoms, the death of nearly one third of patients overall was possibly associated with delayed administration of antitoxin, and the death of nearly two thirds of patients overall may have been associated with delayed diagnosis of diphtheria (*31*,*32*). By comparison, 2 fatal cases in Japan occurred in women 57 and 66 years of age who had respiratory symptoms; 1 death may have been attributable to delay in administration of antitoxin, and the other death may have been because the patient was diagnosed with *C. ulcerans* infection too late. 

When we compared cases of *C. ulcerans* infection in the United Kingdom and Japan in terms of deaths, we observed a similar course of death in both countries. Administration of antitoxin is the primary treatment for diphtheria, but because diphtheria antitoxin is a preparation made from horse serum immunized with diphtheria toxin, its administration may be accompanied by adverse events, such as serum sickness. The decision requires judgment in considering the risks and benefits of antitoxin administration. In the United Kingdom and Japan, 94% of patients with *C. ulcerans* infection survive. In both cases, administration of macrolide antibiotics is the main treatment method, whereas diphtheria antitoxin is administered to severely ill patients (*31*,*32*)

When we compared the symptoms caused by *C. ulcerans* infection, we found that ≈80% of patients in the United Kingdom had respiratory symptoms and ≈20% had nonrespiratory symptoms. We noted the same tendency in cases in Japan (respiratory symptoms in 66% of patients and nonrespiratory symptoms in 34%). In Belgium, on the contrary, prevalence of nonrespiratory symptoms were as high as 64%, and respiratory symptoms were observed in 36% of cases (*31*–*33*). The different proportions of respiratory and nonrespiratory symptoms of *C. ulcerans* infection in the 3 countries may be related to the immunologic status of patients with respect to diphtheria toxin. In our study, the patients with respiratory symptoms were mostly elderly and severely ill, whereas the patients with nonrespiratory symptoms were relatively young, and few were severely ill (Table 1). In Japan, young persons have high levels of antibody titers against diphtheria toxin, but this antibody titer declines with age (*35*). Patients with high antibody titers who have *C. ulcerans* may show nonrespiratory symptoms without exacerbation of respiratory symptoms. Different diphtheria toxoid vaccination schedules in the United Kingdom and Belgium may also influence symptoms after *C. ulcerans* infection (*37*,*38*).

We also considered the status of vaccination for *C. ulcerans* infections. The diphtheria vaccine in Japan became available in 1948, and after several changes in the inoculation content, the formulation now in use was implemented in 1995. The current vaccination schedule in Japan is to inoculate 3 times at intervals of 3–8 weeks starting at 3 months of age and to give the fourth inoculation 1 year after the third inoculation. At 11 years of age, children receive a fifth boost and are not vaccinated after that point (*35*). In Japan, to understand the state of immunity to diphtheria toxoid, a certain number of persons are randomly selected from prefectures nationwide and their antibody titers are measured every 4–5 years. That survey is commissioned by the government of Japan and is conducted by NIID and local health authorities. According to those survey data, the proportion of persons in their 50s who still had antibody titers at a level protective against diphtheria decreased to ≈10% (*36*). When we interviewed patients in our study and asked about their vaccination status, most of the patients >60 years of age, except for the 20-year-old patient in case 13 and the 6-year-old patient in case 14, so vaccination status was usually unknown. In addition, the average age of patients in our study with severe respiratory symptoms was 67 years (Table 2), and patients born before 1948 had not been vaccinated. We hypothesize that persons in the older age group are inadequately vaccinated or unvaccinated against diphtheria toxin and that the characteristics of *C. ulcerans* infection are related to the vaccination system in Japan. 

According to reports on *C. ulcerans* infection in the United Kingdom and Belgium, many patients, especially those who died or were severely ill, were unvaccinated or inadequately vaccinated (*31*–*33*). The vaccination schedule in the United Kingdom is to inoculate 4 times until 5 years of age; at the age of 14, persons receive a fifth booster and are not vaccinated after that point (*37*). The vaccination schedule in Belgium is to inoculate 6 times until at the age of 14–16 years. Adults in Belgium are recommended to be vaccinated with a diphtheria toxoid–containing vaccine every 10 years (*38*). When we compared the diphtheria toxoid vaccination schedule in the United Kingdom and Belgium with Japan, we found that the United Kingdom schedule is very similar to that of Japan, but the Belgium schedule is similar to the WHO-recommended schedule and the US Advisory Committee on Immunization Practices schedule and includes vaccination for adults. This difference may explain why the number of patients with *C. ulcerans* infection in Belgium has remained constant over the past decade or so (*38*).

In light of those findings, it appears that *C. ulcerans* infections tend to affect generations with reduced levels of diphtheria antitoxin antibodies. Moreover, because the risk for severe disease from *C. ulcerans* infections increases with age, we recommend that adults be vaccinated with diphtheria toxoid vaccine to prevent the spread of this infection. In fact, the European Union (which includes Belgium), the US Centers for Disease Control and Prevention, and WHO recommend that adults be vaccinated with a diphtheria toxoid–containing vaccine every 10 years after completing the initial vaccination series in childhood (*1*,*19*,*35*,*38*).

Among the limitations of this research, we summarized clinical data on 34 cases of *C. ulcerans* infection reported in Japan over a 20-year period, and we reported the clinical features, treatments performed, and prognoses of these cases. However, not all cases were captured, and some data on the reported cases may have been incomplete, which may affect the reliability of our findings. Therefore, it is necessary to verify our findings with more case information in the future. 

In Japan, diphtheria caused by diphtheria toxin–producing *C. diphtheriae* occurred in ≈100,000 patients around 1945, and ≈10% of them died. This form of diphtheria was significantly reduced by regular vaccination with the diphtheria toxoid vaccine, and the last such case was reported in 2000 (*34*). Meanwhile, *C. ulcerans* infections have been increasing over the past 20 years and have replaced disease cases caused by *C. diphtheriae*. Clinicians and various local hygiene agencies have been alerted to this kind of infection. However, the law does not require all cases to be reported. Given the increased number of cases revealed in our study and the WHO position of considering *C. ulcerans* infections to be diphtheria, we suggest that all *C. ulcerans* cases should be included with the infections that currently must be reported immediately.

Appendix Additional information about clinical characteristics of *Corynebacterium ulcerans* infection, Japan.
