# Supplementary material for: Clinical Characteristics of Corynebacterium ulcerans Infection, Japan
Source: Emerg Infect Dis. 2023 Aug;29(8):1505–15. doi: 10.3201/eid2908.220058 (PMC10370844; doi:10.3201/eid2908.220058)
Supplement: Appendix — Additional information about clinical characteristics of Corynebacterium ulcerans infection, Japan. [file 22-0058-Techapp-s1.pdf]

Article DOI: https://doi.org/10.3201/eid2908.220058

# Clinical Characteristics of *Corynebacterium ulcerans* Infection, Japan

## Appendix

**Appendix Table.** Summary of each case of *C. ulcerans* infection

| Case | Date of onset | Age, y* | Sex* | Symptoms (severity) | Relationship with animals* | Outcome |
|---|---|---|---|---|---|---|
| 1 | Feb. 2001 | 52 | F | Respiratory (severe) | Breeding† cats | Survived |
| 2 | Oct. 2002 | 54 | M | Respiratory (mild) | | Survived |
| 3 | Sep. 2005 | 58 | M | Nonrespiratory (parotid gland swelling (moderate) | Breeding dogs | Survived |
| 4 | Oct. 2005 | 50 | M | Respiratory (moderate) | Breeding cats | Survived |
| 5 | Jul. 2006 | 57 | F | Respiratory (severe) | Parakeet breeding | Deceased |
| 6 | Jan. 2009 | 57 | F | Respiratory (mild) | Breeding cats | Survived |
| 7 | Jul. 2010 | 55 | M | Nonrespiratory (axillary abscess (mild) | Breeding cats | Survived |
| 8 | Oct. 2010 | 51 | F | Respiratory (moderate) | Breeding cats | Survived |
| 9 | Apr. 2011 | 57 | F | Respiratory (moderate) | Breeding cats | Survived |
| 10 | Dec. 2011 | 38 | F | Nonrespiratory (right elbow abscess (moderate) | Breeding cats | Survived |
| 11 | Jan. 2012 | 33 | M | Nonrespiratory (axillary abscess (mild) | Contact cats | Survived |
| 12 | Nov. 2013 | 71 | F | Respiratory (moderate) | Breeding cats | Survived |
| 13 | Apr. 2014 | 20 | F | Respiratory (mild) | Breeding cats | Survived |
| 14 | Apr. 2015 | 6 | F | Nonrespiratory (cervical lymph node abscess (moderate) | Breeding cats | Survived |
| 15 | Sep. 2015 | 66 | F | Respiratory (severe) | Breeding cats | Survived |
| 16 | Feb. 2016 | 17 | F | Nonrespiratory (sole abscess ulcer (moderate) | Breeding dogs | Survived |
| 17 | Mar. 2016 | 63 | F | Respiratory (severe) | Breeding cats | Survived |
| 18 | Mar. 2016 | 66 | F | Respiratory (severe) | Breeding cats | Deceased |
| 19 | May 2016 | | | Nonrespiratory (purulent lymphadenitis of the right neck (mild) | Breeding dogs | Survived |
| 20 | Jul. 2017 | 64 | F | Nonrespiratory (sole skin ulcer, subcutaneous abscess (mild) | Breeding cats | Survived |
| 21 | Feb. 2017 | 62 | F | Respiratory (moderate) | Breeding cats | Survived |
| 22 | Jan. 2017 | 67 | F | Respiratory (severe) | Breeding cats | Survived |
| 23 | Mar. 2017 | 83 | M | Respiratory (moderate) | Breeding cats | Survived |
| 24 | Mar. 2017 | 73 | M | Respiratory (severe) | Breeding cats | Survived |
| 25 | Nov. 2017 | 24 | M | Nonrespiratory (mandibular abscess (moderate) | Contact cats | Survived |
| 26 | Jan. 2018 | 72 | F | Respiratory (severe) | Breeding cats | Survived |
| 27 | Mar. 2018 | | | Respiratory (mild) | Breeding cats | Survived |
| 28 | Jul. 2018 | 73 | M | Nonrespiratory (thigh abscess (moderate) | Breeding cats | Survived |
| 29 | Jul. 2018 | 72 | F | Respiratory (severe) | Breeding cats | Survived |
| 30 | Jan. 2019 | | | Nonrespiratory (*C. ulcerans* isolation in blood cultures of patients with malignant lymphoma, septic shock, ICU management (mild) | Parakeet breeding | Survived |
| 31 | Feb. 2019 | 65 | M | Respiratory (mild) | Breeding cats | Survived |
| 32 | May. 2019 | 36 | M | Respiratory (mild) | Breeding cats | Survived |
| 33 | Dec. 2019 | 72 | F | Respiratory (severe) | Breeding cats | Survived |
| 34 | Dec. 2019 | 78 | M | Respiratory (moderate) | Breeding cows | Survived |

*Missing data for cases: age (n = 3), sex (n = 3), relationship with animals (n = 1).
†The term “breeding” indicates that the patient or their family is raising animals. Whether the animal is kept indoors or outdoors depends on the mindset of the community and its family, but at least indicates that the patient was in contact with the animal.
